# Unveiling the Determinants of Prehospital Delay in Patients With Acute Myocardial Infarction: A Cross-Sectional Study

**DOI:** 10.1155/nrp/7096059

**Published:** 2025-09-28

**Authors:** Wenman Lv, Xin Jin, Yue Yang, Yinji Jin

**Affiliations:** ^1^School of Nursing, Yanbian University, Yanji, Jilin, China; ^2^Department of Ultrasound, TEDA International Cardiovascular Hospital, Tianjin, China

**Keywords:** acute myocardial infarction, prehospital delay, risk factors, symptom

## Abstract

**Objective:** This study aims to comprehend the current status of prehospital delay among patients with acute myocardial infarction. It analyzes the correlation between various factors and prehospital delay and explores the influencing factors.

**Methods:** A cross-sectional survey was conducted. From February to June 2023, 260 AMI patients were selected by consecutive sampling from the study hospital in Yanji City, Jilin Province. General Data Questionnaire, Pain level Scale, Family Support Scale, Psychological distress scale, and Chinese version of the perceived impairment of medical decision-making scale were used. SPSS 28.0 and AMOS 28.0 were employed for *t*-test, chi-square test, Pearson correlation analysis, binary logistic regression analysis, and model construction.

**Results:** The median prehospital delay time was 4.67 h. There were 174 patients with prehospital delay, accounting for 66.92%. The structural equation model indicated that the total effect values of prehospital delay influencing factors from strong to weak were pain level (−0.294), a perceptual disorder of medical decision-making (0.209), psychological distress (0.084), and family support (−0.068).

**Conclusions:** Approximately two-thirds of patients experience a prehospital delay. Risk factors for prehospital delay include being female, lower family monthly income, lower education level, complications, symptom relief after taking medicine, lack of health care awareness, seeing a doctor alone, psychological distress, and perceptual disorder of medical decision-making. Protective factors are the pain level and family support.

**Patient or Public Contribution:** No patient or public contribution.

**Reporting Method:** The authors adhered to the EQUATOR network guidelines STROBE to report observational cross-sectional studies.

**Summary**

• What is already known?
◦ Timely reperfusion therapy within 3–6 h of symptom onset is crucial for acute myocardial infarction patients.
◦ Symptom misinterpretation, lack of healthcare access, and familial roles worsen delays, especially for women and those in rural areas. However, the understanding of the complex relationship between influencing factors, especially in certain populations like those in China, is incomplete.
• What this paper adds?
◦ Specific risk and protective factors for prehospital delays are identified, including gender, income, education, comorbidities, symptom relief after medication, healthcare awareness, transportation mode, psychological distress, impaired medical decision-making, pain level, and family support.
◦ The study finds that pain level is a protective factor, while impaired decision-making and psychological distress are risk factors. It also emphasizes the importance of family support in reducing prehospital delays, which can guide the development of targeted interventions for acute myocardial infarction patients in China and similar populations.

## 1. Introduction

Cardiovascular disease (CVD) is an important health problem facing the world, and the incidence of CVD in China is increasing year by year [1]. The Quality Control Report of China Chest Pain Center in 2021 points out that the incidence of acute myocardial infarction (AMI) in China will continue to increase in the future due to the aging of the population and the increased risk of disease [2], and it is estimated that the number of AMI patients in China will reach 23 million in 2030 [3]. AMI is a common disease, which is characterized by acute onset, rapid deterioration, and high mortality. It is a clinical syndrome in which the blood supply of specific coronary arteries is cut off, resulting in ischemia or hypoxia, and then myocardial destruction in vascular areas [4, 5]. Among them, ST-segment elevation myocardial infarction is the most common disease, which leads to the death of urban and rural residents in China [6].

As the largest developing country in the world, the number of people dying from AMI in China is increasing every year [6]. It is reported in the interpretation of the key points of China Cardiovascular Health and Disease Report 2021 that the mortality rate of AMI is generally on the rise from 2002 to 2018 [1]. Data show that the mortality rate of patients with severe myocardial infarction can be as high as 50%–80% [7]. Therefore, it is important to carry out timely and effective intervention and treatment for AMI patients.

In medical care, “treatment time” is an increasingly important aspect, such as AMI, in which myocardial cells begin to die 20–30 min after lack of coronary artery blood supply, and the focus of emergency treatment is reperfusion treatment within the first few hours of symptom onset [8]. Studies have shown that thrombolytic therapy within 1 h of sudden myocardial infarction symptoms can improve the survival rate to 50%; even if thrombolytic measures were taken within 3 h, the survival rate could increase by 23% [9]. Therefore, this study focuses on the prehospital time of patients.

However, many factors lead to the delay of prehospital time. When the pain is mild, it is easy for patients to ignore the severity of the disease. For example, patients with myocardial infarction complicated with diabetes, if accompanied by neurological diseases, often do not feel severe pain due to decreased sensory function [10], so that patients do not go to the hospital in time [11]. On the family side, some studies have shown that if patients fail to seek help from relatives and friends in time, they cannot get help effectively the first time, which may lead to a delay in prehospital medical treatment [9]. Psychologically, AMI patients will be affected by emotional, cognitive, and behavioral reactions after symptoms occur, such as worries or pessimism, which may be related to night symptoms, heavy workload, and great economic pressure, and finally prolong patients' hesitation time [12].

**Figure 1:**
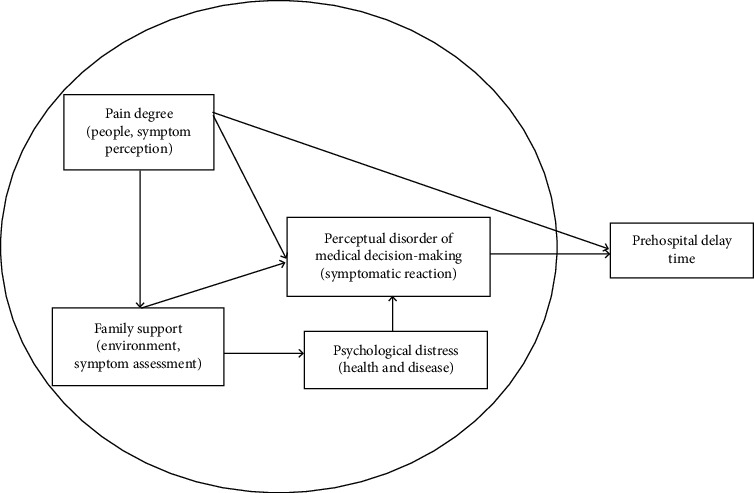
Conceptual framework of this study.

This study is a cross-sectional study, so we focus on the prehospital delay (PHD) of patients with AMI and use the symptom experience and nursing dimensions (people, health and disease, and environment) of symptom management theory as the theoretical framework of this study, and establish the research model as follows (Figure 1).

## 2. Methods

### 2.1. Study Design

This is a cross-sectional study, which uses the consecutive sampling method.

### 2.2. Participants and Samples

From February 2023 to June 2023, patients with AMI hospitalized in the Department of Cardiology of a top-three hospital in Yanji City, Jilin Province research setting. The sample size is determined by 5–10 times the selected variables and then expanded by 20% considering the missing or invalid questionnaires. There are 21 variables in this study, and the calculation formula of sample size is 21 × 10 × (1 + 20%), so the estimated sample size is 252 patients.

During this period, 270 questionnaires were distributed and 260 valid questionnaires were recovered, with an effective rate of 96.3%.

Patients included in the study must meet the following conditions: (1) meet the diagnostic criteria of the 2019 Guidelines for the Diagnosis and Treatment of Acute ST-segment Elevation Myocardial Infarction, and are diagnosed as patients with AMI by clinicians; (2) adults aged ≥ 18 years old; (3) the condition of the subject is in a stable stage; (4) informed consent and voluntary participation in this study. Patients with mental illness, unstable clinical conditions, or unconsciousness during the prehospital period are excluded.

### 2.3. Instruments

#### 2.3.1. Clinical Variables

To collect general demographic information, we used a questionnaire, including 16 items, including patients' age, sex, BMI, monthly income per family, marital status, educational level, medical payment method, working status, and smoking history, are there any complications, whether percutaneous coronary intervention (PCI) is performed, whether symptoms are relieved after taking medicine, whether they are aware of health care at ordinary times, whether they are transferred to hospital, how to see a doctor, and the time spent from symptom onset to arrival at hospital.

#### 2.3.2. Pain Level

Pain level was determined using numerical rating scale (NRS), also known as digital analogy table and 11-point digital score method. This method requires patients to describe pain intensity with 11 numbers 0–10, 0 is painless, 1–3 is mild pain, 4–6 is moderate pain, 7–9 is severe pain, and 10 is severe pain [13]. NRS is a digital intuitive expression method, and patients only need to use numbers to express the level of pain they feel when symptoms occur.

#### 2.3.3. Family Support

The family support scale was used to evaluate the level of family support received by patients. The scale was designed by Procidano and Heller [14] in the United States to measure family support for patients' spirit, emotion, intimacy, and information feedback. The Cronbach's alpha coefficient of the scale was 0.750. In 2011, Yang [15] and scholars revised the scale according to China's national conditions. There are 15 items in this scale, which are evaluated by dichotomy. If you answer “yes,” you will get 1 point, and if you answer “no,” you will get 0 points, and the total score will be 0–15 points. According to the score, it is divided into three grades: 0–5 is divided into low level, 6–10 is divided into medium level, and 11–15 is divided into high level [16]. The Cronbach's alpha coefficient of the scale used in this study was 0.716; it indicates that the scale has acceptable internal consistency reliability in the context of this study and is suitable for the current data analysis.

#### 2.3.4. Psychological Distress

The level of psychological distress of patients was evaluated by a simple psychological status rating scale. There are 10 items in the scale, including the frequency of nonspecific mental health–related symptoms such as anxiety and stress level experienced in the past four weeks. The frequency of each item is divided into five grades: all time, most of the time, sometimes, occasionally, and almost none. When scoring, score 5 points, 4 points, 3 points, 2 points, and 1 point, respectively, for the above five grades. Then, sum the scores of 10 items, and the scores > 15 showed psychological distress, 10–15 showed mild distress, 16–21 showed moderate distress, 22–29 showed severe distress, and 30–50 showed severe psychological distress. [17]. The Cronbach's alpha coefficient of this study was 0.757.

#### 2.3.5. Perceived Impairment of Medical Decision-Making

The Chinese version of the perceived impairment of medical decision-making scale was used to evaluate patients' perceived Chengdu of medical decision-making. The original scale was compiled by Jordanian scholar AL-HASSAN and others [18]. In 2014, Li et al. [19] finally developed the Chinese version of the scale of perceptual impairment in medical decision-making, with Cronbach's alpha coefficient of the scale being 0.74. The scale is a one-dimensional scale with only 10 items and adopts a Likert6 score. The scores from “strongly disagree” to “strongly agree” are 1–6, respectively, and the total score is 10–60. The higher the score, the worse the delay in medical decision-making. Cronbach's alpha coefficient was 0.794 in this study.

To analyze the data, we used IBM SPSS version 28.0 and AMOS 28.0 software. We use frequency and percentage to obtain descriptive statistics of variables. The chi-square test was used for univariate analysis, and then, statistically significant factors were included in multivariate analysis. We use AMOS 28.0 software to construct a hypothetical model, use the maximum likelihood method to estimate parameters, and constantly modify the structural equation model. The alpha standard of the test is set to 0.05.

### 2.4. Ethical Considerations

The study protocol was conducted in strict accordance with the ethical principles outlined in the Declaration of Helsinki and received formal approval (Approval No YBU20220134) from the Institutional Review Board of Y University School of Medicine on 16 December 2022. Written informed consent was obtained from all study participants prior to their inclusion in the research.

## 3. Results

### 3.1. Sample Characteristics

The median PHD time of 260 AMI patients was 4.67 h, the shortest was 30 min and the longest was 30 h. Studies have shown that more than 50% of ischemic myocardium can be prevented from necrosis if myocardial reperfusion is quickly and effectively restored within 3 h of onset [20]. According to the time from the onset of symptoms to the hospital, 260 patients were divided into a delayed group (> 3 h) and a nondelayed group (≤ 3 h), each group contained 174 people and 86 people, respectively. The incidence of PHD in AMI patients was 66.92%.

Among the 260 subjects, 167 (64.2%) were male, 93 (35.8%) were female, 79 (30.4%) had a high school education or above, and 161 (61.9%) were married in marital status. Additional information is presented in Table 1.

Comparing the differences in pain level, family support, psychological distress, and perceptual disturbance of medical decision-making between delayed and nondelayed AMI patients, the results showed that the above four variables were statistically significant (*p* < 0.05), as shown in Table 2 for details.

### 3.2. Univariate Analysis of Pre-Hospital Delay

Combined with Tables 1 and 2, there were significant differences among AMI patients in age, gender, per capita monthly income of families, education level, medical payment method, smoking or not, are there any complications, whether symptoms were relieved after taking medicine, do you have health care awareness at ordinary times, whether to transfer to hospital, the way to the hospital, pain level, family support, psychological distress, and perceptual disorder of medical decision-making (*p* < 0.05).

### 3.3. Multivariate Analysis of Prehospital Delay

#### 3.3.1. Variable Assignment

In this study, the prehospital medical treatment time of AMI patients is divided into a delayed group and a nondelayed group according to whether there is a delay, which belongs to two classification variables. To explore the relationship between AMI patients and independent variables, a classification logistic regression is adopted, and meaningful variables in single-factor analysis are included in the regression equation for statistical analysis.

#### 3.3.2. Binary Logistic Regression Analysis

According to binary logistic regression analysis, gender (odds ratio [OR], 2.732), per capita monthly income of families (OR, 5.036), education level (OR, 3.353), are there any complications (OR, 3.214), the relief of symptoms post medicine intake (OR, 2.831), the presence of ordinary health care consciousness (OR, 2.997), route of hospital (OR, 2.792), pain level (OR, 0.091), family support (OR, 0.696), psychological distress (OR, 1.126), and perceptual disorder of medical decision-making (OR, 2.861) were the influencing factors of PHD (*p* < 0.05). Details are shown in Table 3.

### 3.4. Construction of Structural Equation Model for Influencing Factors of Prehospital Delay

#### 3.4.1. Correlation Analysis of Influencing Factors of Pre-Hospital Delay of Subjects

In this study, Pearson correlation analysis was used to analyze the pain level, family support, psychological distress, perceptual disorder of medical decision-making, and prehospital time. The results showed that prehospital time was negatively correlated with pain level (*r* = −0.270, *p* < 0.01), negatively correlated with family support (*r* = −0.369, *p* < 0.01), positively correlated with psychological distress (*r* = 0.244, *p* < 0.01), and positive correlated with perceptual disorder of medical decision-making (*r* = 0.402, *p* < 0.01), which met the requirements of model construction. See Table A1 for details.

#### 3.4.2. Structural Equation Model of Influencing Factors of Prehospital Delay of Subjects

**Figure 2:**
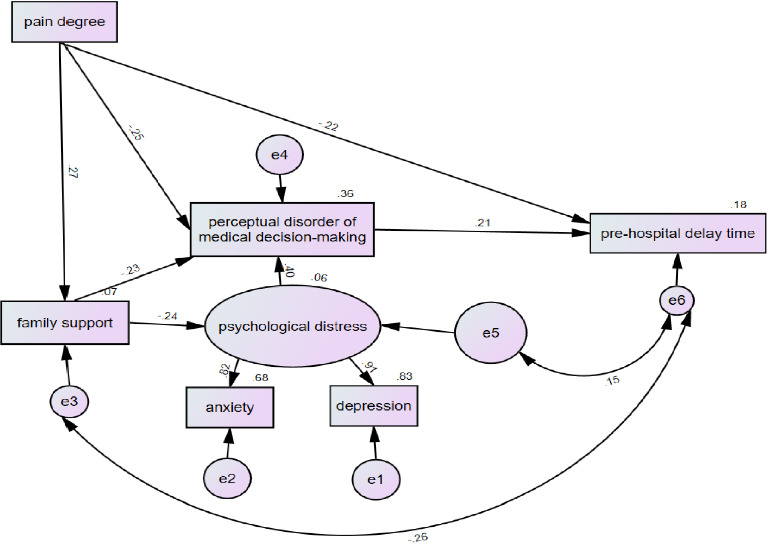
Model M1.

The focus of this study is to explore the impact of pain level, family support, psychological distress, and perceptual disorder on medical decision-making on PHD of AMI patients, so only the above four factors and medical time were included in the fitting structural equation model. According to previous related literature reports and related theoretical research, combined with the above analysis results, the structural equation model M1 of influencing factors of PHD of AMI patients is constructed, as shown in Figure 2. The fitting index results of hypothetical model M1 are shown in Table A2.

#### 3.4.3. Path Analysis Results of Influencing Factors of Prehospital Delay in the M1 Model

The standardized path coefficient analysis results of model M1 are shown in Table 4.

The path analysis results show that

• Path 1: The pain level of AMI patients can have a direct negative impact on the PHD time.
• Path 2: Family support of AMI patients can have an indirect negative impact on PHD time through psychological distress and perceptual disorder of medical decision-making.
• Path 3: The perceptual disorder of AMI patients' medical decision-making can have a direct positive impact on PHD time.

#### 3.4.4. Path–Effect Relationship of Factors Influencing Pre-Hospital Delay in the M2 Model

The total effects of the absolute value of the influencing factors of a PHD from strong to weak were pain level (−0.294), the perceptual disorder of medical decision-making (0.209), psychological distress (0.084), and family support (−0.068). Bootstrap estimation method was used, the sample size was 5000, and the confidence interval was 95%. The results showed that all the path relationships in the M2 model were valid, as shown in Table 5 for details.

## 4. Discussion

This study divides the subjects into a delayed group (174 patients) and a nondelayed group (86 patients) based on a 3-h threshold [21], and the incidence of PHD was 66.92%. In this study, the median delay time of AMI patients was 4.67 h, which was higher than the 2.17 h studied by Huang and other scholars [22], and 2.2 h in Australia and New Zealand [23], similar to 4 h in Argentina and Brazil [23], and lower than 11.5 h in Bangladesh [24]. The median time of PHD in different studies was different in different countries. The possible reasons are analyzed as follows: (1) in previous studies, AMI patients were grouped according to whether ST segment changes occurred in their hearts, and different disease types corresponded to corresponding disease symptoms and severity, which indirectly affected the pre-hospital medical treatment time of patients; (2) related to the research site, the research site in Bangladesh is two research institutes; (3) different studies have different definitions of delay time and exclusion criteria. For example, Huang scholars [22] took patients whose time from onset to treatment was less than and equal to 24 h as inclusion criteria.

This study finally identified 11 influencing factors of PHD in AMI patients, including gender, per capita monthly income of families, educational level, are there any complications, whether symptoms were relieved after taking medicine, do you have health care awareness at ordinary times, the way to the hospital, psychological distress, and perceptual disorder of medical decision-making; protective factors include pain level and family support.

### 4.1. Risk Factors

#### 4.1.1. Female

The results of this study showed that the incidence of PHD in female patients was significantly higher than that in male patients (*p*=0.015, OR = 2.732), which was consistent with domestic and foreign studies [23, 25, 26]. A study from Pakistan showed that the delay rate of female patients was 35.8%, while that of male patients was 18.7% [27]. A Swedish report showed that the delay time was 1.5 h for women and 1 h for men [28]. It could be seen that women receive reperfusion therapy later than men. The reasons might be as follows: 1. women did not want to bother other family members, because women played the role of caregivers in the family, fearing that once they were hospitalized, no one would take care of the daily life of family members [12], but men rarely had such thoughts [29]. When experiencing pain in areas other than the chest (such as stomach, back, and shoulder pain), women would be delayed for a long time because they believed that the symptoms would disappear or think that the symptoms were not serious [28].

#### 4.1.2. Lower Monthly Income per Family

The results of this study showed that the incidence of PHD in patients with lower income was significantly higher than that in patients with higher income (*p* < 0.001, OR = 5.036), which was consistent with the results of domestic [23] research. The possible reasons were as follows: (1) the proportion of nonworking patients in this study was large, and the decrease in income sources had caused their limited economic bearing capacity; patients might think that when they went to a big hospital for treatment, doctors would use “expensive drugs,” “new drugs,” or expensive instruments for examination, so low-income families might face high costs.

#### 4.1.3. Lower Educational Level

The results of this study showed that the incidence of PHD in patients with lower education levels was significantly higher than that in patients with higher education levels (*p*=0.005, OR = 3.353). Due to the lack of understanding of diseases, some patients had little knowledge of all aspects of surgical treatment such as PCI [30]. The results of the previous study showed that in in-depth interviews with ACS patients when symptoms appeared [31], patients compared to prior experiences and evaluated them with their own knowledge or experience, which was consistent with the conclusion of Wilson and other scholars [32]. However, the results of the evaluation would also be affected by their own educational background, experience, and other factors. In other words, the lower the education level, the less they knew about the disease, which caused the patients to be unable to make an accurate diagnosis at the early stage of their illness, thus delaying the time for medical treatment [33].

#### 4.1.4. Complications

The results of this study showed that the incidence of PHD in patients with complications was significantly higher than that in patients without complications (*p* = 0.006, OR = 3.214), which was consistent with the results of Nielsen and Goedhart [25]. Most previous studies at home and abroad had found that common complications such as diabetes [34], hypertension [35], angina pectoris [36], hyperlipidemia [37], myocardial infarction history [38], cardiogenic shock [39], and so on were all risk factors of PHD, but domestic studies had found that myocardial infarction history had nothing to do with PHD [40]. A predictive model study found [41] that digestive system diseases such as chronic gastritis and gastrointestinal ulcers were also independent risk factors affecting PHD, which had not been included in the analysis in many similar studies. To sum up, the reasons might be as follows: (1) diabetes mellitus: diabetic patients had extensive microvascular lesions in a coronary artery, which led to poor opening of collateral circulation. When infarction occurs, the myocardium is suddenly ischemic and cannot release sufficient metabolites, so there would be no symptoms of angina pectoris [42]. Some studies have found that diabetes often leads to peripheral neuropathy, which leads to a decrease in patients' sensitivity to pain. In addition, the clinical manifestations of hypoglycemia were somewhat similar to AMI, so after AMI, patients were more likely to be mistaken for hypoglycemia, which led to delayed treatment [43]. (2) Hypertension and angina pectoris: patients who might be complicated with hypertension were more likely to have atypical symptoms of AMI [44]. In addition, chronic angina pectoris in STEMI patients with heart failure also made it difficult to distinguish myocardial infarction from chronic chest pain [45]. (3) Digestive system diseases: because the symptoms of myocardial infarction will be covered by pain sites when myocardial infarction occurs, most patients with digestive system diseases will regard it as digestive system disease and relax their vigilance, resulting in delays [46].

#### 4.1.5. Symptoms Are Relieved After Taking the Medicine

The results of this study showed that the incidence of PHD in patients with remission of symptoms after taking medicine was significantly higher than that in patients with no remission of symptoms after taking medicine (*p*=0.012, OR = 2.831), which was consistent with the research of Zhang Yishan and other scholars [47]. In the early stage of AMI, both male and female patients chose to take nitroglycerin, and the frequency of taking nitroglycerin in women was almost twice that in men [28]. Recurrent symptoms of ACS patients were one of the important factors for decision-making delay [48]. Frequent chest pain and similar symptoms often led patients to take medication and rest to relieve their illness based on previous medical experience [49].

#### 4.1.6. Lack of Health Care Awareness at Ordinary Times

The results of this study showed that the incidence of PHD in patients with a lack of health care awareness was significantly higher than that in patients with health care awareness (*p*=0.007, OR = 2.997), which was consistent with the research results of Guo Jinjin [50], Guo Jingjie [51], Liang Baixue [52], and other scholars. This paper analyzed the possible reasons for this result: Some people hesitated or gave up the physical examination directly because of the cumbersome physical examination items, which further aggravated the phenomenon of delayed medical treatment [53]. However, physical examination was closely related to patients' health awareness and professional awareness of their diseases. The more times of physical examinations, the more professional guidance they received from medical staff, which was conducive to the improvement of patients' disease compliance. Moreover, they had a strong concept of medical treatment and had less impact on delaying medical treatment [52].

#### 4.1.7. See a Doctor by Yourself

The results of this study showed that the incidence of PHD of patients who came to the hospital by themselves was significantly higher than that of patients who called ambulances (*p*=0.021, OR = 2.792), which was consistent with the research of Hu Danqing and other scholars [23]. A foreign study showed that the average delay time of transporting patients by ambulance is 45 min, and the average delay time of transporting patients by oneself was 97 min [54]. The hospitalization delay time of outpatients was more than 5 times that of emergency patients. If patients go to the emergency department as soon as possible, the hospitalization delay time would be significantly shortened [36]. The reasons might be as follows: (1) The selected area in this study was remote in the north, and the economic level was slightly lower than that in the southern cities. In addition, more patients were not working in this study, and it took a certain amount of money to call an ambulance [26]. (2) It still took time to wait after calling an ambulance, but it was more convenient to take a taxi or drive, which was consistent with foreign studies [38]. (3) Some patients did not think that their illness was very serious and did not understand the urgency of reperfusion therapy [55].

#### 4.1.8. High Psychological Distress

The binary logistic regression analysis showed that higher mental distress was the independent risk factor of PHD. The risk of PHD in patients with higher mental distress was 1.126 times higher than that in patients with lower mental distress (*p*=0.001, OR = 1.126). There was a positive correlation between mental distress and PHD (*R* = 0.244, *p* < 0.01). If AMI patients develop depressive symptoms 2 weeks before admission, they will be delayed to receive treatment in the hospital [9]. Li Na and other scholars mentioned two questions in the interview outline of AMI patients: (1) What did you think before going to see a doctor? And how is it done? Can you tell me the whole experience and feelings of going to see a doctor in detail? The patient's answers to these two questions were: “I didn't want to wake my wife in the middle of the night, so I didn't take any measures.” And “This was my third heart stent operation, and I was also afraid.” This showed that the patient had some psychological troubles. The results of this study also showed that the psychological distress of AMI patients was influenced by their own characteristics and environment, which led to different emotional cognition and coping styles. Therefore, we should pay close attention to patients' psychological feelings and reactions at different stages [26].

#### 4.1.9. The Perceptual Disorder of Medical Decision-Making Is High

Univariate analysis and regression analysis showed that higher perceptual impairment of medical decision-making was an independent risk factor for PHD. The risk of PHD of patients with higher perceptual impairment of medical decision-making was 2.861 times that of patients with lower perceptual disorder of medical decision-making (*p*=0.025, OR = 2.861). Every score of perceptual impairment of medical decision-making increased the risk of delayed medical treatment of AIM patients by 2.861 times, and there was a positive correlation between them (*r* = 0.402, *p* < 0.01). The reason was that some patients were afraid of diseases or their own health. They would ask their families before going to the hospital, or use the internet and other channels to know the relevant situation. After learning that surgery might be needed, they would be more afraid.

### 4.2. Protective Factors

#### 4.2.1. The Level of Pain Is High

In this study, binary logistic regression showed that moderate or above pain was a protective factor for PHD of AMI patients, and the risk of PHD of patients with moderate or above pain was 0.091 times higher than that of patients with moderate or above pain (*p*=0.002, OR = 0.091), which was consistent with the research of scholars in Li [56]. All the above studies show that the higher the level of pain patients felt, the more attention they would pay, and seeking medical treatment as soon as possible could reduce the risk of delay. The possible reason was that patients with mild pain were not easy to make a decision to go to the hospital in time because they did not realize the seriousness of the illness [51]. This wrong understanding would only aggravate the illness and delay the treatment of patients.

#### 4.2.2. Family Support Is Higher

In this study, the incidence of PHD in patients with lower family support was significantly higher than that in patients with higher family support (*p* < 0.001), and binary logistic regression analysis showed that higher family support was a protective factor for patients with PHD, and the risk of PHD in patients with higher family support was 0.696 times that of patients with lower family support (OR = 0.696), which was consistent with the research results of Zhou and Xu [57] on patients with acute exacerbation of chronic obstructive pulmonary disease. In China, family is the most important social relationship of individuals, and the most and earliest help individuals get comes from families. Therefore, medical workers should teach patients how to obtain and make good use of family support, encourage patients to establish good communication with their families, and let patients feel the support of their families and gain a sense of security, so that they can treat their illness with an optimistic attitude, thus reducing the time of PHD; In addition, family members of patients should be guided and encouraged to give support to patients [58]. To sum up, it is suggested that medical staff should pay attention to the family support of AMI patients when they carry out PHD intervention.

### 4.3. PHD Action Path of Research Subjects

#### 4.3.1. Direct Path Analysis of Pain Level to PHD

The structural equation model showed that the level of pain had a direct negative impact on the PHD time, with a direct effect value of −0.224 and a total effect value of −0.294, that was, the higher the level of pain, the shorter the pre-hospital medical time. The more serious the pain was, the more attention it would attract from family members. Painless or mild pain would lead to the influence of traditional concepts such as difficult early identification, insufficient attention or unawareness, and reluctance to affect the normal operation of family life because of their health [59]. Therefore, only after experiencing typical symptoms such as severe chest pain [60] can patients find that the disease is serious, pay enough attention to the disease, and then take the initiative to seek medical treatment, which causes delay during this period.

#### 4.3.2. Path Analysis of Family Support to Pre-Hospital Delay

The structural equation model showed that family support could indirectly and negatively affect the PHD time through psychological distress and perceptual disorder of medical decision-making, with an effect value of −0.068. Most elderly patients hold the attitude of “carrying the disease if they can” or they can't help because their children are not around. From this perspective, many scholars took family factors as a whole factor [21] to further explore how family dynamics, communication modes, and the relationship between family members specifically affect patients' responses and decision-making. According to foreign researchers, the possibility of depression and anxiety in patients without family support was between 27% and 60% [61]. It could be seen that if the patient's family atmosphere was tense, the patient would easily have psychological troubles such as helplessness and despair, which would eventually lead to the patient's delay in seeing a doctor.

#### 4.3.3. Path Analysis of Perceptual Impairment of Medical Decision-Making to Pre-Hospital Delay

The structural equation model showed that perceptual impairment of medical decision-making had a direct positive impact on PHD time, with an effect value of 0.209. Different from general demographic factors such as age, patients' perceptual impairment in making medical decisions could be improved by effective intervention measures. Scholar Li et al. [62] provided participants with a video-based and interactive virtual AMI attack experience using narrative intervention, which truly restored various perceptions and social scenes, made patients immersive, carefully rehearsed psychologically in the decision-making process, and evaluated possible problems and problems. Compared with traditional preaching, narrative intervention not only changed patients' willingness not to seek medical treatment but also shared symptom experience in the form of groups, which enhances the effect of peer education [49], relieves patients' negative emotions, and indirectly reflects that narrative intervention would help patients establish awareness of seeking medical treatment in time, thus reducing the delay of medical treatment decision-making.

### 4.4. Research Advantages

Generally speaking, this study used symptom management theory to construct the research hypothesis of this study, which filled the lack of theoretical support in previous studies. In the previous studies, the influencing factors of medical decision-making delay were often explored, and decision-making delay was a part of PHD, the content of this study was more extensive and comprehensive. In terms of research methods, few papers in China discussed the pathway relationship of PHD of AMI patients. By constructing a structural equation model, this study analyzed the pathway relationship between pain level, family support, psychological distress, perceptual disorder of medical decision-making, and PHD behavior in detail, and provided a reference for formulating effective intervention measures.

### 4.5. Research Limitations

This study had three limitations. First of all, the convenience sampling method was used in this study, and only the patients with AMI in a 3A hospital in one area were investigated, but the samples could not fully reflect the overall situation; secondly, this study was a cross-sectional study, which was limited, and it is necessary to further explore experimental research in the future; finally, this study used self-reported subjective measurement to evaluate patients' medical treatment time, which might have recall bias.

### 4.6. Recommendations

By analyzing the mechanism of different paths on PHD, combined with clinical practice and research evidence, this paper puts forward the following suggestions:

#### 4.6.1. Optimize the Prehospital Diagnosis and Treatment Mechanism

In the intervention study, scholars in Liu adopted the WeChat platform to see a doctor. By establishing a collaborative emergency network and using the mobile telemedicine system to transmit patients' relevant information, remote consultation was conducted in time, so as to achieve the purpose of early intervention, rapid diagnosis, and timely transfer [63]. At the same time, grass-roots hospitals should strengthen their own diagnosis and treatment level, strengthen the standardization of emergency management in township hospitals, formulate a sound rural emergency management model and standards, and improve the referral process from grass-roots hospitals to tertiary hospitals [12].

#### 4.6.2. Strengthen Health Education

In society and hospitals, we should educate the typical symptoms of AMI and the appropriate emergency treatment methods through various forms such as WeChat Official Account, broadcast audio, and offline lectures [12]. The medical staff in the community service center should develop a risk scoring system to evaluate the cumulative risk of high-risk groups of AMI in the community and screen out high-risk groups [64]. It is suggested that the family should intervene from the perspective of health literacy and form family-based health education in the early stage of AIM, so as to improve the patients' and their families' knowledge about how to prevent and treat AMI. Once the symptoms occur, they should call an ambulance as soon as possible and transport them to the hospital quickly [65].

## 5. Conclusion

The PHD time of the subjects needed to be further shortened. The risk factors of PHD include female, lower monthly income per family, lower education level, complications, symptoms are relieved after taking the medicine, lack of health care awareness at ordinary times, seeing a doctor by yourself, psychological distress, and perceptual disorder of medical decision-making; protective factors include pain level and family support.

Consequently, targeted policies and guidelines should prioritize implementing tailored health education for high-risk groups to improve symptom recognition and emphasize delay risks, enhancing family support mechanisms, and establishing efficient referral pathways for individuals experiencing psychological distress or decision-making difficulties. Addressing these modifiable factors is crucial to effectively reduce PHD times and improve patient outcomes.

## Figures and Tables

**Table 1:** Comparison of the difference in prehospital delay in general data (*n* = 260).

Variables	Classifications	Delay group (*n* = 174)	Nondelayed group (*n* = 86)	*χ* ^2^	*p*
Age (years)	< 60	51 (29.3)	39 (45.3)	6.541	0.011
	≥ 60	123 (70.7)	47 (54.7)		

Gender	Male	96 (55.2)	71 (82.6)	18.788	< 0.001
	Female	78 (44.8)	15 (17.4)		

BMI	< 18.5	16 (9.2)	6 (7.0)	1.081	0.782
	18.5–23.9	47 (27.0)	20 (23.3)		
	24.0–27.9	98 (56.3)	54 (62.8)		
	≥ 28.0	13 (7.5)	6 (6.9)		

Per capita monthly income of families (yuan)	< 3000	90 (51.7)	29 (33.7)	7.515	0.006
	≥ 3000	84 (48.3)	57 (66.3)		

Marital status	Married	101 (58.0)	60 (69.8)	3.354	0.067
	Others	73 (42.0)	26 (30.2)		

Education level	Junior high school and below	128 (73.6)	53 (61.6)	4.177	0.041
	High school/junior college or above	46 (26.4)	33 (38.4)		

Medical payment method	Urban/urban and rural medical insurance	155 (89.1)	83 (96.5)	4.103	0.043
	At one's own expense	19 (10.9)	3 (3.5)		

Working state	On-the-job	44 (25.3)	28 (32.6)	1.519	0.218
	Inactive	130 (74.7)	58 (67.4)		

Smoking	Yes	108 (62.1)	41 (47.7)	4.874	0.027

Complications	Yes	124 (71.3)	55 (64.0)	4.919	0.027

PCI	Yes	95 (54.6)	56 (65.1)	2.615	0.106

The relief of symptoms post medicine intake	Yes	42 (39.5)	34 (24.1)	6.596	0.010

The presence of ordinary health care consciousness	Yes	55 (31.6)	40 (46.5)	5.512	0.019

Transfer to hospital	Yes	112 (64.4)	42 (48.8)	5.749	0.017

Route of hospital	Self-visit	139 (79.9)	58 (67.4)	4.854	0.028
	Ambulance	35 (20.1)	28 (32.6)		

Abbreviations: BMI = body mass index, PCI = percutaneous coronary intervention.

**Table 2:** Comparison of the differences in prehospital delay in various related factors (*n* = 260).

Variables	Delay group (*n* = 174)	Nondelayed group (*n* = 86)	*t*/*χ*^2^	*p*
Pain level (score)	5.75 ± 2.86	6.84 ± 1.72	3.233	0.001
Painless	14 (8.0)	0 (0.0)	21.215	< 0.001
Mild pain	29 (16.7)	3 (3.5)		
Moderate pain	45 (25.9)	33 (38.4)		
Severe pain	69 (39.7)	45 (52.3)		
Severe pain	17 (9.7)	5 (5.8)		
Family support (score)	6.49 ± 3.12	9.69 ± 2.97	7.910	< 0.001
Low family support	68 (39.1)	7 (8.1)	38.371	< 0.001
Medium family support	87 (50.0)	48 (55.8)		
Advanced family support	19 (10.9)	31 (36.1)		
Psychological distress (score)	30.56 ± 5.52	24.46 ± 6.61	−5.271	< 0.001
Mild distress	1 (0.6)	7 (8.1)	26.706	< 0.001
Moderate distress	9 (5.2)	14 (16.3)		
Severe perplexity	71 (40.8)	40 (46.5)		
Severe psychological distress	93 (53.4)	25 (29.1)		
Perceptual disorder of medical decision-making (score)	41.71 ± 7.40	34.19 ± 10.38	−6.713	< 0.001

**Table 3:** Results of binary logistic regression analysis of influencing factors of prehospital delay in AMI patients.

Independent variable	Value	OR	95% CI		*p*
			Lower	Upper	
Gender	Female	2.732	1.214	6.146	0.015
Per capita monthly income of families (yuan)	< 3000 = 1	5.036	2.147	11.813	< 0.001
Education level	Junior high school and below	3.353	1.455	7.727	0.005
Complications	Yes	3.214	1.403	7.365	0.006
The relief of symptoms post medicine intake	Yes	2.831	1.253	6.399	0.012
The presence of ordinary health care consciousness	Yes	2.997	1.344	6.680	0.007
Hospital of route	Self-visit	2.792	1.171	6.653	0.021
Pain level	Painless and mild pain	0.091	0.020	0.408	0.002
Family support (score)		0.696	0.605	0.802	< 0.001
Psychological distress (score)		1.126	1.050	1.209	0.001
Perceptual disorder of medical decision-making (score)		2.861	1.142	7.167	0.025

*Note:* Hosmer–Lemeshow test = 0.830; *R*^2^ = 0.564.

**Table 4:** Path coefficient analysis results of influencing factors of median delay in the M2 model.

Dependent variable	Independent variable	Nonnormalized coefficient	Normalization coefficient	S.E.	C.R.	*p*
Family support < --pain level		0.351	0.266	0.079	4.440	< 0.001
Psychological distress < -family support		−0.194	−0.236	0.057	−3.420	< 0.001
Perceptual disorder of medical decision-making < -pain level		−0.909	−0.253	0.190	−4.787	< 0.001
Perceptual disorder of medical decision-making < -family support		−0.622	−0.228	0.150	−4.143	< 0.001
Perceptual disorder of medical decision-making < -psychological distress		1.330	0.400	0.196	6.782	< 0.001
Depression < --psychological distress		1.041	0.910	0.110	9.436	< 0.001
Anxiety < --psychological distress		1.000	0.822			
Prehospital delay time < --pain level		−0.427	−0.224	0.117	−3.664	< 0.001
Prehospital delay time < --perceptual disorder of medical decision-making		0.111	0.209	0.037	3.030	0.002

*Note:* S.E. denotes standard error, and C.R. is the critical ratio, that is, the *t* value.

**Table 5:** Effect relationship of influencing factors of prehospital delay (standardized value).

Path	Effect relation	Effect value	Deviation correction 95% CI	
			Lower limit	Upper limit
Pain level ⟶ prehospital delay time	Total effect	−0.294	−0.424	−0.154
	Direct effect	−0.224	−0.362	−0.077
	Indirect effect	−0.070	−0.119	−0.035

Family support ⟶ prehospital delay time	Total effect	−0.068	−0.113	−0.034
	Direct effect	—	—	—
	Indirect effect	−0.068	−0.113	−0.034

Psychological distress ⟶ prehospital delay time	Total effect	0.084	0.040	0.148
	Direct effect	—	—	—
	Indirect effect	0.084	0.040	0.148

Perceptual disorder of medical decision-making ⟶ prehospital delay time	Total effect	0.209	0.103	0.318
	Direct effect	0.209	0.103	0.318
	Indirect effect	—	—	—

**Table 6:** Correlation analysis of influencing factors of prehospital delay in AMI patients (*n* = 260, *r*).

Variable	Pain level	Family support	Psychological distress	Perceptual disorder of medical decision-making	Prehospital delay time
Pain level	1				
Family support	0.266^∗∗^	1			
Psychological distress	0.081	−0.237^∗∗^	1		
Perceptual disorder of medical decision-making	−0.297^∗∗^	−0.395^∗∗^	0.408^∗∗^	1	
Prehospital delay time	−0.270^∗∗^	−0.369^∗∗^	0.244^∗∗^	0.402^∗∗^	1

^∗∗^ *p* < 0.01.

**Table 7:** Test results of fitness degree of model M1.

Model	*χ* ^2^/df	GFI	AGFI	RMSEA	NFI	IF	CFI
Standard (acceptable)	< 5	> 0.70	> 0.70	< 0.10	> 0.70	> 0.70	> 0.70
Standard (excellent)	< 3	> 0.90	> 0.90	< 0.08	> 0.90	> 0.90	> 0.90
Fitting index	2.749	0.986	0.928	0.082	0.974	0.984	0.983

## Data Availability

The data supporting the findings of this study are available from the corresponding author by sending an email (kimeunhee1004@gmail.com) upon reasonable request.
